# Development of high-throughput quantitative analytical method for l-cysteine-containing dipeptides by LC–MS/MS toward its fermentative production

**DOI:** 10.1186/s13568-019-0817-2

**Published:** 2019-06-21

**Authors:** Yusuke Kawano, Maeka Shiroyama, Koji Kanazawa, Yasushi A. Suzuki, Iwao Ohtsu

**Affiliations:** 1 Graduate School of Life and Environmental Sciences, University of Tsukuba, Tsukuba, Japan; 2 Microbiology Research Center for Sustainability, University of Tsukuba, Tsukuba, Japan; 3 Euglena Co., Ltd, Tokyo, Japan; 4 Biochemical Laboratory, Saraya Co. Ltd, Kashiwara, Japan

**Keywords:** Cysteine-containing dipeptides, *Escherichia coli*, Sulfur metabolism, Ultraperformance liquid chromatography–tandem mass spectrometry

## Abstract

l-Cysteine (Cys) is metabolically fundamental sulfur compound and important components in various cellular factors. Interestingly, free-form Cys itself as a simple monomeric amino acid was recently shown to function in a novel antioxidative system (*cysteine*/*cystine shuttle system*) in *Escherichia coli*. However, as for Cys-containing dipeptides, the biological functions, effects, and even contents have still remained largely elusive. The potential functions should be a part of cellular redox system and important in basic and applied biology. For its progress, establishment of reliable quantitation method is the first. However, such accurate analysis is unexpectedly difficult even in Cys, because thiol compounds convert through disulfide-exchange and air oxidation during sample preparation. Addressing this problem, in this study, thiol molecules like Cys-containing dipeptides were derivatized by using monobromobimane (thiol-specific alkylating reagent) and detected as *S*-bimanyl derivatives by liquid chromatography coupled to tandem mass spectrometry (LC–MS/MS). Sample separation was processed with a C18 column (2.1 mm × 150 mm, 1.7 μm) and with water-acetonitrile gradient mobile phase containing 0.1% (v/v) formic acid at flow rate of 0.25 ml/min. The mass spectrometer was operated in the multiple reaction monitoring in positive/negative mode with electrospray ionization. The derivatization could indeed avoid the unfavorable reactions, namely, developed the method reflecting their correct contents on sampling. Furthermore, the method was successfully applied to monitoring Cys-containing dipeptides in *E. coli* Cys producer overexpressing *bacD* gene. This is the first report of the quantitative analysis of Cys-containing dipeptides, which should be useful for further study of fermentative production of Cys-containing dipeptides.

## Introduction

For all organisms, sulfur is an essential element like carbon and nitrogen, although its elemental composition is basically much low (Ingenbleek and Kimura 2013). Sulfur-containing proteinogenic amino acids are l-cysteine (Cys) and l-methionine (Met). Unlike mammals, most microorganisms can biosynthesize Cys and Met from environmental inorganic sulfur sources like sulfate (SO_4_^2−^), thiosulfate (S_2_O_3_^2−^), sulfite (SO_3_^2−^), sulfide (S^2−^), etc. Many other organic sulfur compounds, most of which are metabolized from Cys, also play biologically critical roles especially in cellular redox reactions; e.g., catalytic residue of enzymes, iron-sulfur cluster, coenzymes, glutathione, etc. In addition to such well-understood roles as sulfur supplier, cellular Cys was recently found to function in a novel antioxidative system in *Escherichia coli*, designated as “cysteine/cystine shuttle system” (Ohtsu et al. 2010, 2015). In this system, Cys is exported from the cytoplasm to the periplasm by the membrane transporter YdeD, then detoxifies H_2_O_2_ by converting it to H_2_O as reducing equivalents there. An alternatively generated oxidized product l-cystine (a Cys dimer via disulfide bond) is imported back to the cytoplasm by the membrane transporter FliY-YecSC to regenerate Cys for its continuous recycling without de novo Cys biosynthesis. In this way, free-form Cys itself as a simple monomeric amino acid exhibits a unique function in a cellular biology.

To shift a view to dimeric amino acid (dipeptide), many dipeptides itself have also been known to exert various biological effects such as antihypertensive effect (Arg-Phe, Ile-Trp) (Enari et al. 2008; Kagebayashi et al. 2012), anxiolytic-like effect (Tyr-Leu) (Kanegawa et al. 2010), sedative effect (Ser-His, Ile-His) (Tsuneyoshi et al. 2008), salt taste enhancing effect (Met-Gly, Pro-Gly) (Kino and Kino 2015; Kino et al. 2016), and so on. Focusing on Cys-containing dipeptides, those like Cys-Glu, Cys-Ser, Cys-Try, etc. exert inhibitory effect against tyrosinase (Tseng et al. 2015). However, biological functions, effects, and even contents of Cys-containing dipeptides have still remained largely elusive. It is envisaged that the unidentified function of Cys-containing dipeptides should be somewhat similar but somehow different to monomeric Cys, and should be a variety of important ones in both basic and applied biology. Thus, currently unrealized technique of fermentative production of Cys-containing dipeptides should be also important. In this context, accurate and easy-to-handle analytical method to quantitate Cys-containing dipeptides is required firstly. Such accurate analysis is actually difficult even in Cys. This is because sulfur compounds with thiol group (R-SH), which is qualitatively redox-active, naturally convert through chemical reactions such as disulfide-exchange and oxidation by air oxygen during the preparation. Hence, even recently prevailing metabolomic analysis is in fact considered to be inadequate for a strict measurement of thiol molecules.

Regarding such a problem, we are recently developing and improving analytical method to quantitate sulfur metabolites including Cys (Kawano et al. 2017; Tanaka et al. 2019; Yamada et al. 2019; Nakajima et al. 2019), based on the preceding leading-edge researches (Ida et al. 2014; Kawano et al. 2015; Nakano et al. 2015; Ohmura et al. 2015). In this method, thiol molecules like Cys, homocysteine, glutathione, sulfite, sulfide, thiosulfate, etc. in the sample are chemically derivatized by using monobromobimane (thiol-specific alkylating reagent) simultaneously with the metabolite extraction, and they are detected and quantitated as bimane-derivatives by liquid chromatography coupled to tandem mass spectrometric (LC–MS/MS) analysis. The derivatization should enable to avoid such unfavorable and extra chemical redox reactions in thiol molecules, thereby rendering the method feasible to reflect their correct contents on the sampling time point. This methodological strategy should be expansively applicable to Cys-containing dipeptide analysis, although it has not yet been undertaken. In this study, we thus attempted to establish high-throughput quantitative analytical method for l-cysteine-containing dipeptides by LC–MS/MS utilizing the bimane-derivatization technique, and indeed achieved it successfully.

## Materials and methods

### Bacterial strains, plasmids, and oligonucleotides

Bacterial strains and plasmids used are listed in Table 1. *E. coli* K-12 BW25113 (Baba et al. 2006) was used as wildtype (WT) in this study, and was supplied by the National BioResource Project-*E. coli* at the National Institute of Genetics in Japan. Molecular biology techniques such as gene cloning, DNA manipulation, transformation of *E. coli* strains etc., were basically performed according to standard methods (Sambrook et al. 1989). For the construction of the plasmid pQE85-BsBacD, a coding region of *bacD* gene was amplified from the genomic DNA of *Bacillus subtilis* 168 (Takagi et al. 1997) by PCR, using a primer pair of 5′-agtCATATGgagagaaaaacagtattggtc-3′ and 5′-actGGATCCtcatactggcagcacatactt-3′ containing *Nde*I and *Bam*HI restriction site as capitalized, respectively. The resultant PCR product was digested with both *Nde*I and *Bam*HI, and ligated into vector part of pQE85 (the derivative of pQE80L [QIAGEN] and a kind gift from Dr. Tohru Dairi and Dr. Yasuharu Satoh) digested with the same enzymes. Introducing pQE85-BsBacD (with ampicillin resistant cassette) carrying *lacI* into *E. coli*, cloned *bacD* gene could be overexpressed under T5 promoter with *lac* operator by IPTG induction. As for a plasmid pCys^HP^ (with tetracycline resistant cassette), the detail is in the reference (Tanaka et al. 2019). Briefly, this plasmid allows *E. coli* cells to overproduce Cys and is the pACYC184 derivative carrying *serA* (T410 stop), *ydeD* and *cysE* (T167A, G203S, T234S, P252L, M256Q) originally derived from *E. coli* but mutated under the control of *ompA* promoter of constitutive expression type.

**Table 1:** Bacterial strains and plasmids used

Strains or plasmids	Genotype	References or sources
Strains		
*Escherichia coli* BW25113	*rrnB3 ∆lacZ4787 hsdR514 ∆(araBAD)567 ∆(rhaBAD)568 rph*-*1*	Baba et al. (2006)
*Bacillus subtilis* 168	*trpC2*	Takagi et al. (1997)
Plasmids		
pACYC184	Tet^R^, Cm^R^	Nippon Gene
pCys^HP^	pACYC184 with *serA* (T410 Stop), *ydeD* and alterd *cysE* (T167A, G203S, T234S, P252L, M256Q) genes under the control of the *ompA* promoter, Tet^R^	Lab strain^a^ Tanaka et al. (2019)
pQE85	Amp^R^, a derivative of pQE80L (Qiagen)	This study
pQE85-BsBacD	pQE85 with *bacD* gene from *Bacillus subtilis*	This study

^a^The plasmid pDES (with *serA* [T410 Stop], *ydeD* and altered *cysE* [T167A] genes) from ajinomoto is first supplied (Wiriyathanawudhiwong et al. 2009). However, this plasmid was accidentally obtained in our laboratory during 10 years of stock

### LC–MS/MS experiment

**Fig. 1:**
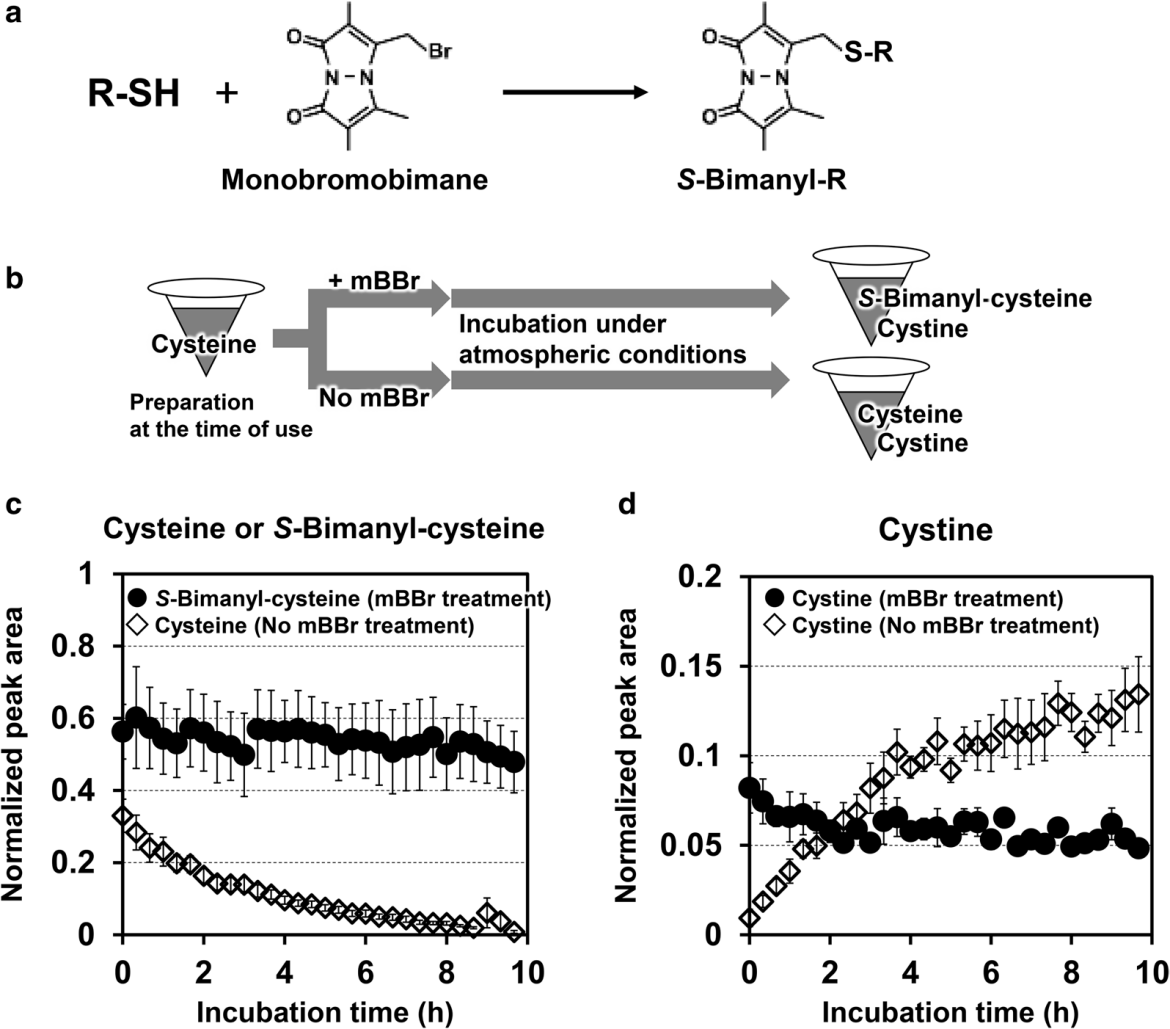
Spontaneous chemical conversion of cysteine by reacting with atmospheric oxygen to produce cystine and effect to avoid the conversion by the treatment of chemical derivatization of thiol group of cysteine utilizing monobromobimane. **a** Reaction of thiol molecule and monobromobimane. **b** Schematic representation of the experimental procedures. Briefly, cysteine aqueous solution prepared at the time of use was immediately treated by adding mBBr solution (denoted as “+ mBBr”) or only its solvent (denoted as “No mBBr”). The solutions were incubated under atmospheric conditions and then intermittently subjected to the measurement by LC–MS/MS analysis (see “Materials and methods”). **c** Time course of cysteine-bimane content in “mBBr treatment” solution (closed circle) and cysteine content in “No mBBr treatment” solution (open diamond). **d** Time course of cystine content in “mBBr treatment” solution (closed circle) and “No mBBr treatment” solution (open diamond). The contents were determined as the peak area in mass chromatogram monitoring *m/z* characteristic of individual target compounds, and are represented as the normalized peak area after normalization with that of internal standard. Data values are mean ± SE (n = 3)

For the experiment in Fig. 1, 100 μl of 13 μM Cys prepared at the experiment, 10 μl of 50 μM d-camphor-10-sulfonic acid sodium salt (CSA, internal standard), and 10 μl of 200 mM Tris–HCl pH8.8 were mixed. Into the solution, 10 μl of 1.3 mM monobromobimane (mBBr) in dimethyl sulfoxide (mBBr treatment) or only dimethyl sulfoxide (No mBBr treatment) was added, and 1 μl of the solution was immediately subjected to analysis of LC–MS/MS (Nexera UHPLC system with on-line LC–MS 8040; Shimadzu). The sample was kept at 25 °C, and LC–MS/MS analysis was repeated every 20 min until 10 h, as previously reported (Kawano et al. 2015). The target quantity levels were determined from the peak area of mass chromatograms, monitoring each *m/z* characteristic to the individual target, and were represented as normalized peak are after normalization with that of the internal standard (CSA).

**Fig. 2:**
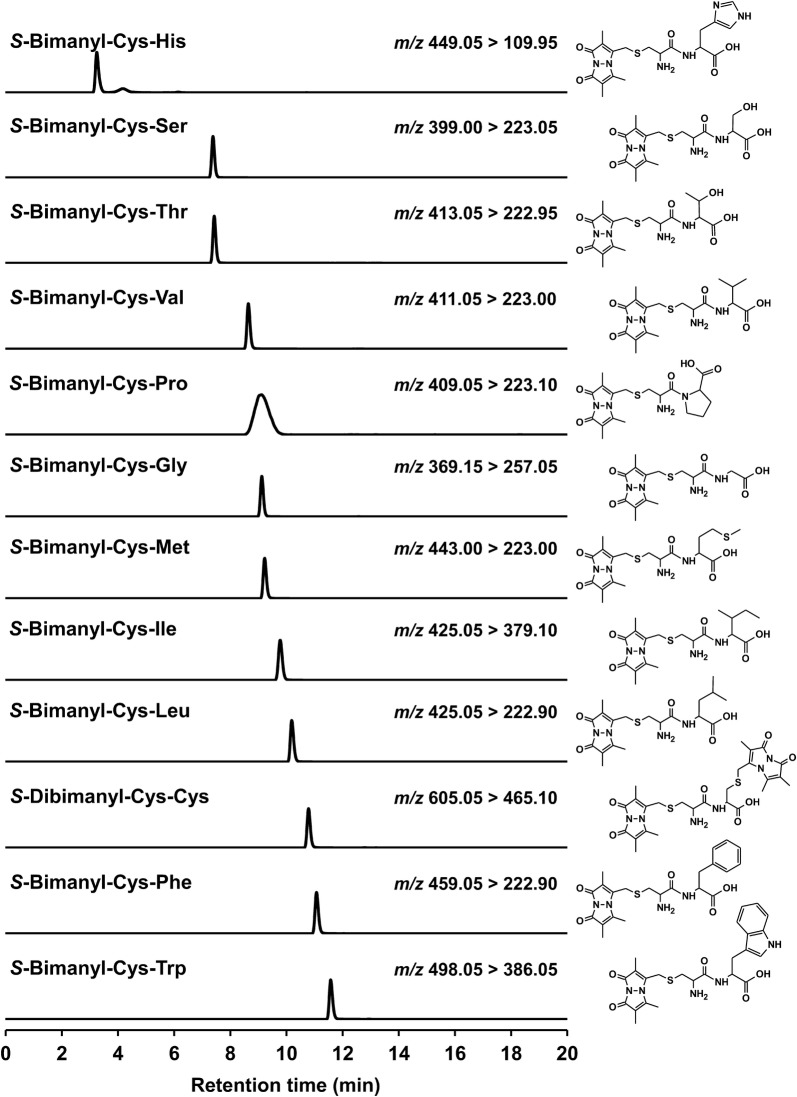
Mass chromatograms of cysteine-containing dipeptide authentic standards derivatized with monobromobimane. The y-axis (not shown) represents normalized intensity of ion count in the mass chromatogram monitoring *m/z* characteristic of individual target compounds, x-axis represents time in the analysis (0–20 min). The *m/z* transitions and structural formulae of each *S*-bimanyl derivative are indicated in the chromatogram

In the experiment in Fig. 2, each 70 μl of 100 μM authentic dipeptide (Cys-His, Cys-Ser, Cys-Thr, Cys-Val, Cys-Gly, Cys-Met, Cys-Ile, Cys-Leu, Cys–Cys, Cys-Phe, Cys-Trp, and Cys-Pro; Sigma Aldrich, custom peptide synthesis), 2 μl of 100 mM ammonium hydrogen carbonate, and 0.14 μl of 100 mM mBBr in dimethyl sulfoxide was mixed and incubated for 10 min at room temperature. Into the solution, 30 μl of 0.1% (v/v) formic acid in acetonitrile was added. As previously performed (Kawano et al. 2015), by utilizing this samples, auto-exploration program was carried out and determined the *m/z* transition parameters optimal for the detection of respective bimane-derivatives. Also, the retention time was experimentally investigated from the mass chromatography program monitoring the determined *m/z* in multiple-reaction monitoring mode, permitting the simultaneous detection of multiple targets.

### SDS-PAGE analysis for confirmation of BacD expression

*Escherichia coli* strains of WT pQE85-BsBacD pCys^HP^ and WT pQE85 (a negative control vector with tetracycline resistant cassette) pCys^HP^ (Tanaka et al. 2019) was utilized. The cell cultures were started to exhibit OD_562_ of 0.1 in Terrific Broth medium including 100 μg/ml ampicillin and 10 μg/ml tetracycline at 30 °C with vigorous shaking for 16 h. At 3 h, 0, 0.05, 0.1, 1, and 5 mM IPTG (final conc.) was added to induce BacD expression. The cells were centrifugally collected and washed once with 100 mM Tris–HCl (pH 8.0) buffer. The cell pellet was resuspended with the same buffer. The cells were disrupted on ice with 10 cycles of 40-s sonication and 40-s interval (Handy Sonic model UR-20P, Tomy Seiko), whose output power was set to a maximum. The lysate was centrifuged at 15 krpm at 4 °C for 10 min, and the total protein concentration of the supernatant was determined by general Bradford assay. SDS-PAGE was performed by standard method (separation gel; 10%) applying the supernatants corresponding to 7 μg of the total protein.

### Cell culture, sample preparation, and in vitro Cys-containing dipeptide formation assay of BacD

*Escherichia coli* strains of WT pQE85-BsBacD pCys^HP^ and WT pQE85 pCys^HP^ (Tanaka et al. 2019) was utilized. The cell cultures were started to exhibit OD_562_ of 0.1 in Terrific Broth medium including 100 μg/ml ampicillin and 10 μg/ml tetracycline at 30 °C with vigorous shaking for 16 h. At 3 h, 0.1 mM IPTG (final conc.) was added to induce BacD expression. The cells were centrifugally collected and washed once with 100 mM Tris–HCl (pH 8.0) buffer. The cell pellet was resuspended with disruption buffer (100 mM Tris–HCl [pH 8.0], 0.5 mM 4-(2-aminoethyl)benzenesulfonyl fluoride hydrochloride [serine protease inhibitor], and 10% [w/v] glycerol) to give the ratio of 5 ml of the buffer to 1 g (wet weight) of the cells. The cells were disrupted as mentioned in SDS-PAGE analysis. The lysate was centrifuged at 15 krpm at 4 °C for 10 min, and the supernatant was stored at − 80 °C after the measurement of total protein concentration by general Bradford assay. The in vitro assay solution was prepared by mixing 12.8 μl of Milli-Q water, 5 μl of 200 mM ATP, 5 μl of 50 mM Cys, 5 μl of 50 mM each of amino acids (including Cys), 1 μl of 1 M MgSO_4_, 7.2 μl of 1 M Tris–HCl (pH8.0). After its preincubation, the reaction was started by adding 14 μl of the prepared supernatant, and was kept at 37 °C. The final concentrations of the total protein were 88 μg/μl (in samples from both strains) and of the Tris–HCl (pH8.0) was totally 100 mM. At each time point (0 [just after beginning reaction], 1, 3, and 5 min), 5-μl aliquot of the mixture was sampled and transferred to the derivatization buffer for enzyme inactivation and simultaneous bimane-derivatization of the thiol molecules—the derivatization buffer consists of 49.5 μl of methanol, 0.5 μl of 500 μM CSA, 5 μl of 2 M Tris–HCl (pH8.8), 1 μl of 100 mM mBBr in dimethyl sulfoxide, and 4 μl of dimethyl sulfoxide (totally 60 μl). The resulting solution was well mixed for 10 min. After centrifugation at 15 krpm at 4 °C for 3 min, 40 μl of the supernatant was mixed with 40 μl of Milli-Q water. After centrifuging again, 10 μl of the supernatant was applied for the analysis of LC–MS/MS developed as mentioned above.

## Results

### Features of thiol derivatization by using monobromobimane in its quantitation

In our recent study for measuring biological sulfur compounds, we are applying a method chemically modifying its thiol-group by using monobromobimane (mBBr) (Fig. 1a) before LC–MS/MS analysis. As its benefit, naturally occurring chemical redox reactions during metabolites extraction to a measurement, which is unsuitable in terms of quantitation, should be avoidable. To verify this effect experimentally, we examined time course of quantitative dynamics of Cys and its bimane-derivative, Cys-bimane, in the aqueous solution under atmospheric conditions (Fig. 1b). As the result, in the no mBBr treatment, Cys content was gradually decreased in a time-dependent manner to the level less than 10% of the beginning after 8 h incubation (Fig. 2c), and cystine was inversely increased correspondingly (Fig. 2d). By contrast, in mBBr treatment, Cys-bimane and cystine kept almost constant level throughout the monitored period, respectively (Fig. 2c, d). These results show that bimane-derivatization method is effective to quantitate Cys and also other thiol molecules including Cys-containing dipeptides, probably because it can actually avoid oxidative reaction of Cys into cystine mediated by atmospheric oxygen in the sample preparation.

### Development of analytical method for quantitating Cys-containing dipeptides by LC–MS/MS

Taking advantage of the derivatization to correctly quantitate thiol-compounds, we here attempted to develop quantitation method for Cys-containing dipeptides as its bimane-derivatives by LC–MS/MS. For this purpose, we first determined the *m/z* transition parameters of tandem MS/MS operation in the respective bimane-derivatized Cys-containing dipeptides (Fig. 2, structural formulae) prepared using the authentic compounds and mBBr. Compound registration to the mass analysis were performed according to the procedures that is recommended by the manufacturer. The LC condition (mobile phases, its gradient program, column, etc.) was fundamentally the same as the previous work to measure sulfur metabolites including bimane-derivatives like Cys-bimane, homocysteine-bimane, glutathione-bimane, sulfide-dibimane, sulfite-bimane, thiosulfate-bimane, etc. (Kawano et al. 2015). The retention time was actually investigated using the prepared bimane-derivatives as well. As a result, 12 species of Cys-X-bimane exhibited a single peak in their mass chromatogram monitoring the determined *m/z*. By integrating each detection program, simultaneous detection method of Cys-containing dipeptide was successfully established for 20-min method. The peak of the chromatogram, retention time and *m/z* transition are shown in Fig. 2.

### Validity of quantitative analytical method of the cysteine-containing dipeptides by LC–MS/MS

We here assessed whether the constructed analytical method for Cys-containing dipeptides can be practically utilized in the experiments for biological research. To this end, we constructed *E. coli* mutant strain to biosynthesize Cys-containing dipeptides, harboring the two plasmids (Table 1). One is pQE85-BsBacD carrying *bacD* gene encoding l-amino acid α-ligase derived from *Bacillus subtilis*. The enzyme is known to catalyze the formation of multiple combinations of dipeptide from the various kinds of monomeric amino acids in an ATP-dependent manner (Tabata et al. 2005). The other is pCys^HP^, which is shown to provide Cys overproduction ability (Tanaka et al. 2019).

**Fig. 3:**
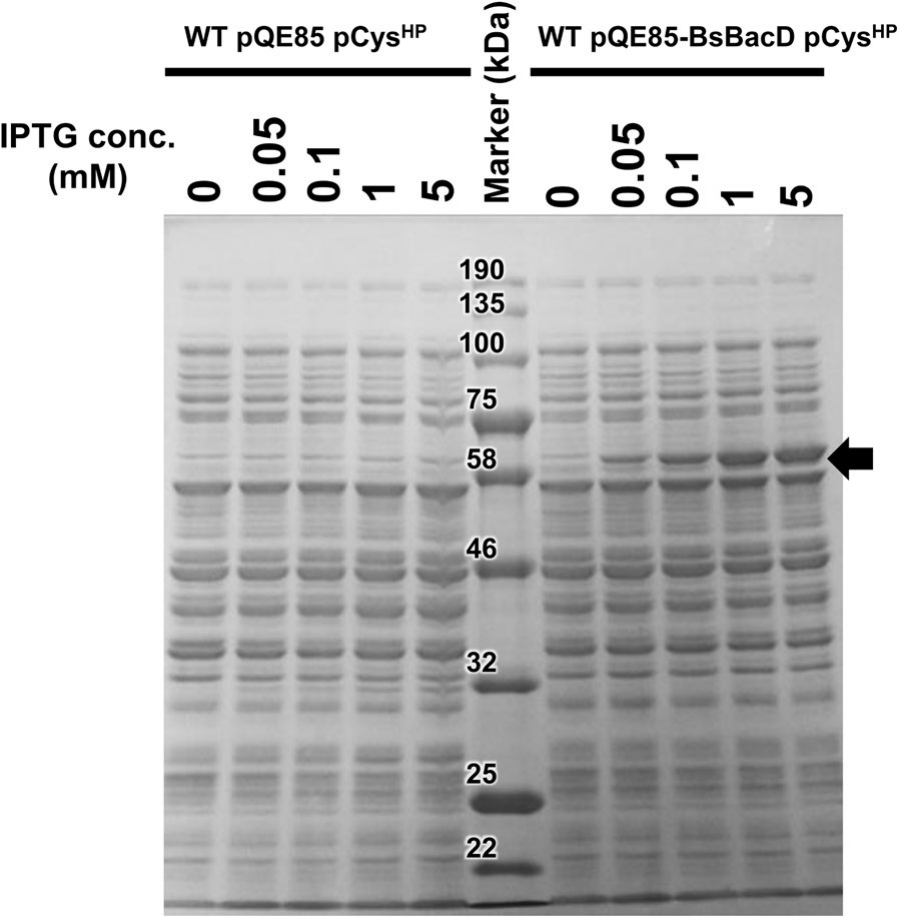
SDS-PAGE analysis for confirmation of BacD expression. The supernatant (7 μg of the total protein) of cell lysate in WT pQE85 pCysHP and WT pQE85-BsBacD pCysHP was analyzed by SDS-PAGE. The protein band considered to be expressed BacD was shown by black arrow. The cell cultures for 16 h with Terrific Broth media were performed adding different IPGT concentrations (0, 0.05, 0.1, 1, and 5 mM [final concentrations]) at 3 h and it is indicated above. Protein marker size (kDa) was indicated above. For details, see “Materials and methods”

We investigated expression level of BacD protein by SDS-PAGE, using the supernatant of the lysate of cells cultivated inducing BacD expression in various IPTG concentrations. As a result, in WT pQE85-BsBacD pCys^HP^ cells, BacD protein (52.3 kDa) was confirmed to be overproduced as soluble protein in the IPTG concentration dependent manner (Fig. 3, arrow). By contrast, the corresponding protein was not observed in WT pQE85 pCys^HP^ cells in all IPTG concentrations.

**Fig. 4:**
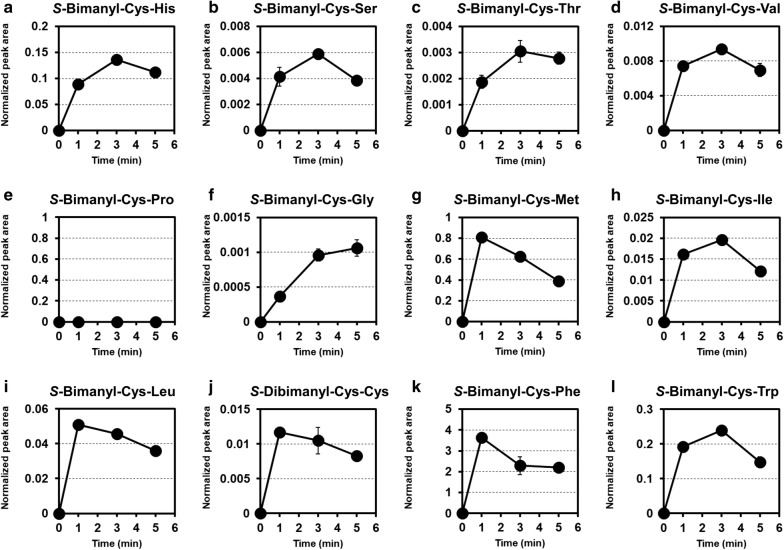
Cysteine-containing dipeptide formation in the in vitro assays measured by the method of *S*-bimanyl derivatization developed in this study (Fig. 2). For the assays, supernatant of cell lysate from WT pQE85-BsBacD pCysHP was utilized, and the substrates were cysteine and **a** histidine, **b** serine, **c** threonine, **d** valine, **e** proline, **f** glycine, **g** methionine, **h** isoleucine, **i** leucine, **j** cysteine, **k** phenylalanine, or **l** tryptophan. Measured *S*-bimanyl dipeptides are indicated in the figures. The y-axis represents normalized peak area in the mass chromatogram monitoring *m/z* characteristic of individual target compounds, and x-axis represents reaction time of the assay; sampling time is 0, 1, 3, 5 min (see “Materials and methods”). Data values are mean ± SE (n = 3)

Preparing the supernatant of the lysate of cells cultivated inducing BacD expression by IPTG addition, we carried out in vitro dipeptide formation assay of WT pQE85-BsBacD pCys^HP^ (Fig. 4). As the substrate combinations, Cys and 12 species of amino acids (including Cys) were tested. Consequently, in the 11 kinds of the combinations, time-dependent formation of bimane-derivatives of the corresponding Cys-containing dipeptides were observed successfully. In the case of substrate combination of Cys and Pro, its assuming product *S*-bimanyl-CysPro was not detected. On the other hand, in the same assay performed using the supernatant of WT pQE85 pCys^HP^ (BacD-absent negative control strain), no dipeptide was formed at all time points in all 12 combinations of substrates, strongly supporting a consistency of the developed assay.

## Discussion

In this study, we developed high-throughput quantitative analysis for biogenic Cys-containing dipeptides by LC–MS/MS (Fig. 2). The method adopts a thiol-derivatization process prior to the measurement. It can exclude the chemical conversion of thiol compounds through redox reactions such as disulfide-exchange and oxidation by oxygen existing under the atmospheric conditions (Fig. 1) in the sample during the preparation for measurements. Namely, this method can provide contents correctly reflecting the state of biological sample on the sampling. We also illustrated that this method is indeed functional and useful for the experiment in the Cys-containing dipeptide formation (Fig. 4).

From results shown in Fig. 1c, another merit of *S*-bimanyl derivative method is considerable. Comparing the normalized peak area of *S*-bimanyl-Cys in the mBBr treatment with that of Cys in the no mBBr treatment at initial time point, the normalized peak area is approximately twofold higher in *S*-bimanyl-Cys than in Cys. This indicates that *S*-bimanyl derivative method is high sensitivity assay compared to direct detection method. This tendency is not limited to Cys but often confirmed in several other thiol compounds (not shown). The probable reason is that *S*-bimanyl derivatization enhances ionization efficiency in MS process, possibly due to a characteristic of *S*-bimanyl derivative moiety. In addition, *S*-bimanyl derivatization is favorable for the detection of too small molecules unsuitable for MS detection, e.g., inorganic sulfur molecules, by increasing mass of the target molecule. Furthermore, although non-ionic and volatile compounds like (off-)flavor molecules consisting of thiol group is basically difficult to sensitively detect in electrospray ionization (ESI) mass spectrometry, the *S*-bimanyl derivatization should allow them to be detected by converting it to nonvolatile ones (not shown). Importantly, this *S*-bimanyl derivatization method is practical and useful method for many scientists, because it requires just widely used type of LC–MS machine (ESI-QqQ) and adopts well-established reverse-phase chromatography system giving easy-to-handle and stable measurements. This method will be effectively developed to increase targets of new sulfur metabolites. Such exploration and identification of unknown sulfur compounds are also developing as *S*-omics utilizing not widely prevalent but state-of-the-art LC–MS machine with a sophisticated strategy (Nakabayashi and Saito 2017; Nakabayashi et al. 2013).

In the previous literature with respect to BacD activities (Tabata et al. 2005), Cys-containing dipeptide formation for Cys-His, Cys-Ser, Cys-Thr, Cys-Met, Cys-Ile, Cys-Leu, Cys–Cys, Cys-Phe, and Cys-Trp was suggested, but Cys-Val, Cys-Gly, and Cys-Pro was not. Similarly, using the same enzyme of BacD, we also showed the activities for Cys-His, Cys-Ser, Cys-Thr, Cys-Val, Cys-Gly, Cys-Met, Cys-Ile, Cys-Leu, Cys–Cys, Cys-Phe, and Cys-Trp, but not for Cys-Pro (Fig. 4). Comparing these two results, of 12 Cys-containing dipeptides, 10 dipeptides were commonly confirmed to be formed (Cys-His, Cys-Ser, Cys-Thr, Cys-Met, Cys-Ile, Cys-Leu, Cys–Cys, Cys-Phe, Cys-Trp) or to be not formed (or formed but less than detection limit) (Cys-Pro). For other 2 dipeptides (Cys-Val and Cys-Gly), we here found to be formed for the first time. It is of note that the assay here just used the supernatant of cell lysate, while the previous report used the purified enzyme to conclude above. In the previous report, whose detection was performed by HPLC-based fluorescent labeling derivatization, in the case of using cell lysate like this study, the activities of Cys-containing dipeptide formations were not detected, probably due to the sensitivity of the measurement and cellular dipeptide-degrading activity coexisting in the cell lysate. In our method, although we could successfully detect the many Cys-containing dipeptide formations, in the most of them, the product level turned to decrease in the short time (1–5 min) (Fig. 4), and was almost abolished in ~ 1 h (data not shown). This also suggests the effect of strong cellular dipeptide-degrading activity. Altogether, our method is practically validated and steadily developed, basically consistent with the previous study.

Using the developed *S*-bimanyl derivatized quantitation method, we can monitor the correct contents of Cys-containing dipeptides in various biological samples, and it will progress the research with respect to physiological functions and valuable effects of Cys-containing dipeptides especially in aspect of new redox system. Also, it enables us to evaluate fermentative production of Cys-containing dipeptides in genetically engineered *E. coli.* In fact, we are trying this subject as a next step and could preliminary confirm Cys-Ser accumulation in *E. coli* cells of WT pQE85-BsBacD pCys^HP^ in the fermentative cultivation, in which Ser was added (not shown). The production (accumulation) was observed in the cells only just after IPTG induction, but undetected thereafter. This suggests a strong dipeptide-degradation activity in the cell, as mentioned. Therefore, it should be effective to construct the disruptant of multiple peptidase genes in *E. coli*. Also, it should be effective to explore l-amino acid α-ligases with various substrate specificities. In fact, several such enzymes were reported from diverse biological species to date (Arai et al. 2013; Kino et al. 2008, 2009). By using the identified genes and its genetically modified genes to the genetic engineering in *E. coli* overproducing Cys by pCys^HP^, we will develop a fermentative production of Cys-containing dipeptides with cost-effectiveness to achieve the market availability in accordance with a philosophy of applied microbiology and biotechnology. Its attainment will promote the research of its physiological functions and beneficial effects.

## Data Availability

The datasets supporting the conclusions of this article are included within the article.

## Abbreviations

*E. coli*: *Escherichia coli*
Cys: l-cysteine
LC–MS/MS: liquid chromatography coupled to tandem mass spectrometry
Met: l-methionine
WT: wildtype
DNA: deoxyribonucleic acid
PCR: polymerase chain reaction
IPTG: isopropyl β-d-1-thiogalactopyranoside
mBBr: monobromobimane
CSA: d-camphor-10-sulfonic acid sodium salt
SDS-PAGE: sodium dodecyl sulfate–polyacrylamide gel electrophoresis
Tris: tris(hydroxymethyl)aminomethane
ATP: adenosine triphosphate
ESI: electrospray ionization
QqQ: triple quadrupole
